# Is surgery with curative intent feasible in old and very old patients with non-small cell lung cancer? – Experience of a certified lung cancer center over one decade

**DOI:** 10.1007/s00423-026-03995-7

**Published:** 2026-02-26

**Authors:** Julia Zimmermann, Julia Walter, Nicole Samm, Fuad Damirov, Niels Reinmuth, Diego Kauffmann-Gerrero, Rudolf A. Hatz, Amanda Tufman, Christian P. Schneider

**Affiliations:** 1 Division of Thoracic Surgery, LMU University Hospital Munich and Asklepios Lung Clinic Gauting, Munich, Germany; 2 Department of Internal Medicine V, LMU University Hospital, Munich, Germany; 3 Department of Thoracic Oncology, Asklepios Lung Clinic Gauting, Gauting, Germany; 4 Comprehensive Pneumology Centre Munich, German Centre of Lung Research (DZL), Munich, Germany

**Keywords:** Non-small cell lung cancer, Elderly patients, Surgical treatment, Overall survival, Progression-free survival

## Abstract

**Purpose:**

Non-small cell lung cancer (NSCLC) accounts for 80% of all lung cancers and, like most other cancers, is a disease of advanced age. This study analyzed the question of whether surgical treatment for non-small cell lung cancer (NSCLC) in elderly patients is acceptable in all operable stages with curative intent.

**Methods:**

In this retrospective analysis, we used data of all NSCLC patients aged over 60 who underwent lobectomy at the Lung Cancer Centre Munich between 2011–2020 and divided them into four groups in ascending order of age. We performed Kaplan–Meier survival analysis and multivariate Cox regression to compare progression-free survival (PFS) and overall survival (OS) between the age groups. To compare numerical outcomes between the age groups we used analysis of variance (ANOVA), and Chi^2^-test for categorical outcomes.

**Results:**

Of 1680 patients undergoing lobectomy, 1221 met our inclusion criteria. It was found that the length of hospital stay increased with advanced age and was significantly longer in older patients (p < 0.001). Although the older patients had more comorbidities, only cardiac arrhythmias occurred significantly more often in the postoperative phase (*p* = 0.02). Kaplan–Meier survival analysis and multivariate Cox regression analysis showed significantly poorer OS for patients aged over 80 compared to patients aged between 60 to 69. PFS was not significantly associated with age.

**Conclusion:**

Surgery with curative intent is feasible in old and very old patients with NSCLC and the oncological outcome is comparable. However, we recommend individual selection in view of patient age.

**Supplementary Information:**

The online version contains supplementary material available at 10.1007/s00423-026-03995-7.

## Introduction

The population's life expectancy is increasing. This means more and more older patients are requiring treatment for malignant diseases [1–3]. Lung cancer surgery is still the best option for curative treatment, especially in the early stages of non-small cell lung cancer (NSCLC). The most common surgical procedure is pulmonary lobectomy [4]. It is evident that older patients face a predicament: lung cancer occurs more frequently in this group, while the conditions for surgical treatment are less favorable (the peak age at the initial diagnosis of lung cancer is between 80 to 84 for men, and 65 to 74 for women in Germany [5]). Declining physical conditions, especially reduced cardiovascular and lung function, lead to increasing postoperative complications and higher mortality [6–8]. Studies have reported satisfactory results in complication rates and survival in elderly patients having early-stage NSCLC [9–12]. However, in clinical practice, particularly in small centers, lung cancer patients are most likely to receive no surgical therapy with advancing age.

There has undoubtedly been a resurgence of interest in this topic in Germany. A new early screening program for lung cancer will be initiated in April 2026. The program is open to patients between 50 and 75 who have smoked for at least 25 years. Participants must either still be smoking or have quit less than 10 years ago. In order to participate, it is necessary that the individual has had a minimum of 15 pack-years of smoking history.

It is crucial to determine whether surgical treatment of NSCLC with curative intent is acceptable in all operable tumor stages in elderly patients. We also need to determine whether the benefits of curative operation outweigh the potentially high perioperative risk and whether very elderly patients (over 85 years of age) should also undergo surgery, or what criteria must be met for surgery to be considered for very elderly patients.

This retrospective study addresses the questions described above. Therefore, we analyzed all patients aged 60 or older who underwent lobectomy due to NSCLC in all operable stages and divided them into four groups in ascending order of age. Each group covered 10 years, with the exception of those being 80 or older. These patients were divided into groups of 80–84 and ≥ 85. This allowed us to distinguish between older and very old patients.

## Methods

### Study design, patient cohort and data collection

This study is based on a patient analysis following surgical treatment (lobectomy) for diagnosed NSCLC. The data collection is retrospective and extends over one decade. The surgical therapy was performed in the Division of Thoracic Surgery LMU Munich and Asklepios Lung Clinic Gauting.

All patients were staged preoperatively, in accordance to the current guidelines. Patients underwent pre- or intraoperative bronchoscopy, pathological lymph node evaluation and FDG-PET/CT scan. Meanwhile, the method of choice for lymph node evaluation was EBUS-TBNA. Historically, a mediastinoscopy was performed in cases when there was a suspicion of lymph node involvement. Mediastinoscopy is still the method of choice for patients with a clinically positive mediastinum (FDG-PET/CT and/or CT) and a negative EBUS-TBNA for malignancy. Patients with clinical stage ≥ II also receive a cranial MRI [13]. After the preoperative staging all patients were discussed at the specific lung tumor board.

The present study comprised patients undergoing curative treatment whose preoperative tumor stage was T1-4, N0-2, M0-M1 (oligometastatic). A small number of M1 patients with oligometastatic underwent multimodal treatment for isolated brain, adrenal, or bone metastases. All metastases were treated preoperatively or postoperatively with curative intent. Two patients (0.16%) exhibited a questionable cN3 status on preoperative PET-CT. In each case, this involved a solitary PET-positive lymph node that could not be confirmed prior to surgery. The surgical intervention involved resection, followed by systemic therapy with curative intent. These decisions were made on a case-by-case basis, reflecting everyday clinical practice.

The patient population was divided into four age-groups: 60 to 69 years, 70 to 79 years, 80 to 84 years and 85 years or older. We chose the shorter age interval for patients in the age of ≥ 80 to be able to examine the results for the very old patients separately. Patients younger than 60 years were excluded.

The data required for the analysis was taken from the electronic patient files and archive documents. The respective variables can be taken from Table 1 and 2, as well as S1 and S2.

**Table 1:** Patient characteristics of study population

	60 to 69 (*n* = 565)		70 to 79 (*n* = 545)		80 to 84 (*n* = 91)		≥ 85 (*n* = 21)			
	mean	sd	mean	sd	mean	sd	mean	sd	*p*-value	
BMI	26,3	5,0	26,1	4,3	26,7	4,4	25,2	2,5	0,65	
Charlson index	3,3	1,5	4,5	1,7	5,5	1,7	5,3	1,3	< 0.0001	***
spirometry										
VC in %	93,1	17,3	93,7	18,9	96,6	19,2	101,0	19,1	0,22	
FEV1%	79,6	17,8	81,9	18,9	87,1	21,2	96,6	27,5	0,002	**
DLCO%	70,0	19,3	70,8	17,6	75,8	18,7	70,7	18,5	0,09	
blood at staging										
Hemoglobin in g/dl	13,5	1,67	13,4	1,7	13,2	1,5	13,0	1,6	0,05	
Creatinine in mg/dl	1,02	0,62	1,04	0,32	1,23	0,89	1,26	0,34	< 0.0001	***
c-reactive protein in mg/dl	1,48	3,37	1,38	3,99	0,83	1,48	1,42	3,5	0,12	
Leucocytes in G/l	8,13	2,78	8,03	4,60	7,12	1,83	6,82	1,55	0,002	**
blood post OP										
Hemoglobin in g/dl	11,9	1,7	11,7	1,6	11,5	1,5	11,4	1,5	0,23	
Creatinine in mg/dl	0,92	0,89	0,93	0,56	0,97	0,43	0,99	0,23	0,0003	***
c-reactive protein in mg/dl	9,40	7,64	8,50	6,49	8,08	6,26	6,27	6,21	0,03	*
Leucocytes in G/l	12,50	4,26	12,10	4,35	11,10	3,70	10,70	4,69	0,01	*
	*n*	%	*n*	%	*n*	%	*n*	%	*p*-value	
comorbidities										
Myocardial infarction	34	6,0%	31	5,7%	8	8,8%	1	4,8%	0,65	
PAD	49	8,7%	59	10,8%	9	9,9%	3	14,3%	0,52	
COPD	195	34,5%	172	31,6%	23	25,3%	5	23,8%	0,25	
bronchial asthma	26	4,6%	25	4,6%	8	8,8%	1	4,8%	0,38	
moderate to severe kidney insufficiency	19	3,4%	29	5,3%	10	11,0%	1	4,8%	0,02	*
metastatic tumor	6	1,1%	7	1,3%	0	0,0%	0	0,0%	0,81	
coronary heart disease	92	16,3%	111	20,4%	20	22,0%	4	19,0%	0,25	
atrial fibrillation	30	5,3%	67	12,3%	14	15,4%	3	14,3%	< 0.0001	***
arterial hypertension	331	58,6%	360	66,1%	73	80,2%	15	71,4%	0,0003	***
pulmonary hypertension	1	0,2%	9	1,7%	3	3,3%	0	0,0%	0,01	**
fibrosis	10	1,8%	4	0,7%	2	2,2%	0	0,0%	0,30	
prior thoracic surgery	48	8,5%	48	8,8%	10	11,0%	3	14,3%	0,60	

Patient characteristics of lung cancer patients older than 60 years with lobectomy. Means with standard deviation of numerical variables and absolute and relative frequency of categorical variables, *sd* standard deviation, *p-value* probability value, *n* number, *BMI* body mass index, *VC* vital capacity, *FEV1* forced expiratory volume in 1 s, *DLCO* diffusing capacity of the lung for carbon monoxide, *PAD* peripheral arterial disease, *COPD* chronic obstructive pulmonary disease

**Table 2:** Tumor characteristics of study population

	60 to 69 (*n* = 565)		70 to 79 (*n* = 545)		80 to 84 (*n* = 91)		≥ 85 (*n* = 21)			
	mean	sd	mean	sd	mean	sd	mean	sd	*p*-value	
tumor size in cm	3,6	2,5	3,6	2,4	4,1	2,6	3,8	1,9	0,27	
	*n*	%	*n*	%	*n*	%	*n*	%	*p*-value	
T status										
T1	210	37,2%	193	35,4%	33	36,3%	5	23,8%	0,57	
T2	229	40,5%	243	44,6%	32	35,2%	14	66,7%	0,04	*
T3	81	14,3%	85	15,6%	19	20,9%	1	4,8%	0,24	
T4	35	6,2%	19	3,5%	6	6,6%	1	4,8%	0,12	
N status										
N0	404	71,5%	399	73,2%	65	71,4%	16	76,2%		
N1	77	13,6%	65	11,9%	13	14,3%	4	19,0%		
N2	83	14,7%	79	14,5%	13	14,3%	0	0,0%		
N3	1	0,2%	0	0,0%	0	0,0%	1	4,8%	0,0001	***
unknown	0	0,0%	2	0,4%	0	0,0%	0	0,0%		
M status										
M0	542	95,9%	522	95,8%	89	97,8%	20	95,2%		
M1	20	3,5%	20	3,7%	1	1,1%	1	4,8%	0,55	
UICC										
I	309	54,7%	292	53,6%	47	51,6%	11	52,4%		
II	124	21,9%	132	24,2%	19	20,9%	8	38,1%		
III	108	19,1%	99	18,2%	23	25,3%	1	4,8%		
IV	14	2,5%	16	2,9%	1	1,1%	1	4,8%	0,47	
unknown	10	1,8%	6	1,1%	1	1,1%	0	0,0%		
histological type										
ACC	318	56,3%	319	58,5%	58	63,7%	13	61,9%	0,55	
SCC	161	28,5%	156	28,6%	24	26,4%	6	28,6%	0,98	
NEC	62	11,0%	44	8,1%	7	7,7%	2	9,5%	0,37	
other	24	4,2%	26	4,8%	2	2,2%	0	0,0%	0,69	
Lymphovascular										
space involvement										
0	413	73,1%	420	77,1%	70	76,9%	15	71,4%	0,21	
1	131	23,2%	108	19,8%	14	15,4%	6	28,6%	0,20	
unknown	21	3,7%	17	3,1%	7	7,7%	0	0,0%		
Vascular invasion										
0	445	78,8%	443	81,3%	71	78,0%	15	71,4%	0,53	
1	100	17,7%	83	15,2%	12	13,2%	6	28,6%	0,24	
unknown	20	3,5%	19	3,5%	8	8,8%	0	0,0%		

Tumor characteristics of lung cancer patients older than 60 years with lobectomy. Means with standard deviation of numerical variables and absolute and relative frequency of categorical variables, *sd* standard deviation, *p-value* probability value, *T* tumor, *N* node, *M* metastasis, *UICC* union international contre le cancer, *ACC* adenocarcinoma, *SCC* squamous-cell carcinoma, *NEC* neuroendocrine carcinoma

### Ethical approval

This monocentric cohort study was performed in accordance with the Declaration of Helsinki and STROBE regulations, following approval of the Ethics Committee of the Ludwig-Maximilians-University Munich (LMU), Germany (#21–0036).

### Surgery outcomes, complications and survival

The classification described in this study is based on postoperative results. The T and N stages have been derived from the postoperative pathological report. Systematic lymphadenectomy was performed intraoperatively. The lymph node stations were listed individually and classified as N1 and N2. The hilar lymph node stations (lymph node station ≥ 10) were categorized as N1 lymph nodes, while the mediastinal lymph nodes (lymph node station 2–9) were designated as N2 lymph nodes. NSCLC were classified according to the 7^th^ edition of the Tumor Node Metastasis (TNM) staging system and The Union for International Cancer Control (UICC) [14, 15].

We compared the groups, by analyzing the number of assessed lymph nodes during surgery, the length of surgery in minutes, the length of hospital stay after surgery in days (LOS) and the total volume of the resected lung in ml. In addition, we compared the groups regarding the different therapies. Finally, we examined the surgical approach (VATS and thoracotomy) and established whether patients received neoadjuvant or adjuvant chemotherapy and/or radiotherapy.

The complications after surgery included the occurrence of: pneumonia, fistula more than five days, acute respiratory distress syndrome (ARDS), pulmonary embolism (PE), delirium, repeated thoracal puncture or repeated placement of a chest tube, postoperative bronchoscopy, cardiac arrhythmia and need for blood transfusion.

The respective variables can be taken from Table 3, as well as Table S3.

**Table 3:** perioperative Outcomes, multimodal therapy and complications

	60 to 69 (*n* = 565)		70 to 79 (*n* = 545)		80 to 84 (*n *= 91)		≥ 85 (*n* = 21)		
	mean	sd	mean	sd	mean	sd	mean	sd	*p*-value
# of assessed lymph nodes	15,8	7,97	16,1	7,91	16,6	8,77	15,0	8,34	0,58
length of surgery in min	183,0	57,1	180,0	54,3	178,0	59,1	176,0	46,8	0,82
length of hospital stay after surgery in days	13,8	6,6	15,0	7,4	15,0	5,7	15,8	6,6	0,0002***
	*n*	%	*n*	%	*n*	%	*n*	%	*p*-value
approach									
video-assisted thoracoscopic surgery	173	30,6%	187	34,3%	32	35,2%	9	42,9%	
thoracotomy	392	69,4%	358	65,7%	59	64,8%	12	57,1%	0,40
multimodal therapy									
neoadjuvant chemotherapy	44	7,8%	22	4,0%	1	1,1%	0	0,0%	0,007**
neoadjuvant radiotherapy	16	2,8%	8	1,5%	0	0,0%	0	0,0%	0,21
neoadjuvant therapy	50	8,8%	23	4,2%	1	1,1%	0	0,0%	0,001**
adjuvant chemotherapy	92	16,3%	57	10,5%	2	2,2%	0	0,0%	< 0.0001***
adjuvant radiotherapy	38	6,7%	25	4,6%	2	2,2%	0	0,0%	0,17
adjuvant therapy	107	18,9%	70	12,8%	3	3,3%	0	0,0%	< 0.0001***
complications									
pneumonia	72	12,7%	83	15,2%	15	16,5%	3	14,3%	0,57
fistula > 5 days after surgery	44	7,8%	48	8,8%	5	5,5%	2	9,5%	0,73
acute respiratory distress syndrome	1	0,2%	1	0,2%	1	1,1%	0	0,0%	0,34
cardiac arrhythmia	41	7,3%	64	11,7%	13	14,3%	3	14,3%	0,02*

Perioperative outcomes of lung cancer patients older than 60. Means with standard deviation of numerical variables and absolute and relative frequency of categorical variables, *sd* standard deviation, *p-value* probability value, *n* number

### Statistical analysis

The data were presented as mean values with standard deviation (SD), absolute and relative frequencies. We compared numerical outcomes between the age groups using analysis of variance (ANOVA), and categorical outcomes using the Chi^2^-test. We performed multivariate Cox regression to compare PFS and OS between the age groups. They were adjusted by sex, American Society Anesthesiologists risk classification (ASA), Charlson comorbidity Index (CCI), histological type, tumor size, nodal involvement, metastases, adjuvant and neoadjuvant therapy, forced expiratory volume in 1 s (FEV1%), and Hb, creatinine, c-reactive protein (CRP) and leukocytes at staging. OS was defined as the time in months from the date of diagnosis to death from any cause. Cancer-specific mortality was not used; therefore, all-cause mortality served as the endpoint. Patients who were lost to follow-up were right-censored at the date of their last documented contact. PFS was defined as the time in months from diagnosis to the first occurrence of disease progression. Progression was defined as any of the following: local recurrence, nodal recurrence, or systemic progression. Patients without a documented progression event were right-censored at their last follow-up.

Kaplan–Meier survival curves were generated to estimate univariate overall survival (OS) and progression-free survival (PFS). Differences between groups were assessed using the log-rank test.

The data analysis was performed using R Version 4.0.0 and RStudio Version 1.4. Tables and figures were created in RStudio and Microsoft Excel.

## Results

### Patient population and tumor characteristics

Our center performed 1680 lobectomies between 2011 and 2020. After excluding re-lobectomies (*n* = 25), patients with missing information on comorbidities (*n* = 9) and patients younger than 60 (*n* = 424), data from 1221 patients remained for the analysis. The age groups consisted of 565 patients between 60 to 69, 545 patients between 70 to 79, 91 patients between 80 to 84, and 21 patients older ≥ 85 years.

The CCI differed significantly between the age groups, with higher comorbidity burden in patients being 80 or older (*p* < 0.0001).We found significant differences in the comorbidities of moderate to severe kidney insufficiency, atrial fibrillation, arterial hypertension, and pulmonary hypertension. The highest proportion of patients with these comorbidities were found in patients between the ages of 80 to 84 years.

There were also some significant differences in the results from spirometry and blood tests at staging as well as after surgery. FEV1% was significantly lower in younger patients and increased with advancing age. Patients aged 60 to 69 years had a mean FEV1% of 79.6%, patients aged 70 to 79 years had 81.9%, patients aged 80 to 84 years had 87.1%, and patients ≥ 85 years had a mean FEV1% of 96.6% (*p* = 0.002). Creatinine levels differed significantly between the age groups. They increased preoperatively with increasing age from 1.02 mg/dl to 1.04 mg/dl, 1.23 mg/dl, and 1.26 mg/dl (*p* < 0.0001). Similar results were found for creatinine levels after surgery – we found: 0.92 mg/dl in the youngest age group, 0.93 mg/dl in patients aged 70 to 79, 0.97 mg/dl in patients aged 80 to 84, and 0.99 mg/dl in patients over the age of 84 (*p* = 0.0003). Additionally, we found that leucocyte levels were significantly different at staging and after surgery, with higher mean values in the younger patients and lower in the elderly. Mean CRP values after surgery were significantly higher in younger patients than in the elderly and decreased with each age group from 9.4 mg/dl, to 8.5 mg/dl, to 8.08 mg/dl, and 6.27 mg/dl (*p* = 0.03).

We found only minor differences in the tumor properties. Statistically, there were no significant differences in T1-, T3-, T4-status, distant metastases, UICC stage, histological type, vascular invasion or Lymphovascular space involvement. The patient and tumor characteristics are summarized in Tables 1 and 2, as well as in the supplementary Tables S1 and S2.

### Surgical outcomes, perioperative therapy and complications

We found that the LOS in days increased with advancing age and was significantly longer in older patients (13.8 vs. 15.0 vs. 15.0 vs. 15.8, *p* = 0.0002). The length of surgery, numbers of assessed lymph nodes, and total volume of preparation did not differ significantly.

A significantly higher proportion of patients in the younger age groups received neoadjuvant (*p* = 0.001) and adjuvant therapy (*p* < 0.0001). This was mostly driven by higher proportions of neoadjuvant and adjuvant chemotherapy.

With a prevalence of 14.3% of patients in the two highest age groups, cardiac arrhythmia was the only surgical complication that was significantly different between the groups (*p* = 0.02). The surgical outcomes, perioperative therapy and postoperative complications are summarized in Table 3 and supplementary Table S3.

### Kaplan–Meier survival analysis

**Fig. 1:**
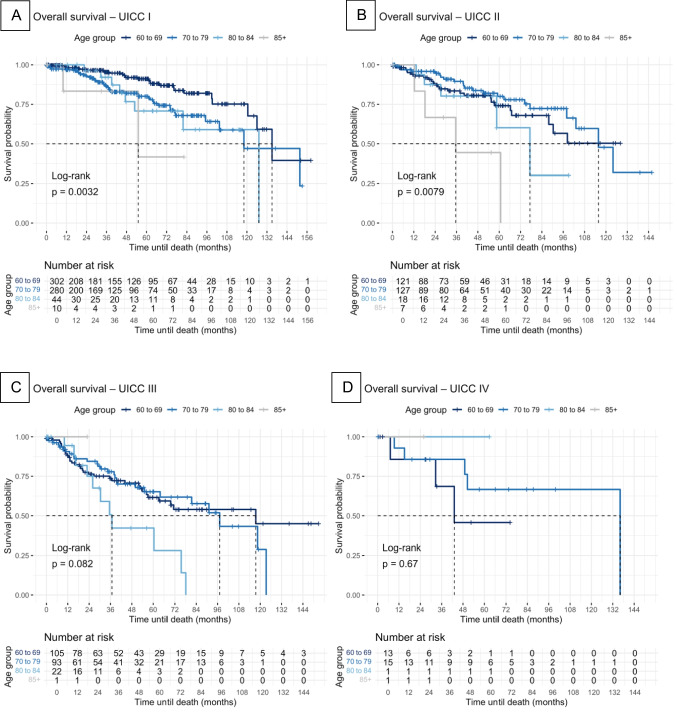
Kaplan–Meier overall survival analysis Kaplan–Meier survival curves were constructed to estimate univariate overall survival (OS). Differences between groups were assessed using the log-rank test. OS was analyzed for the different age groups in the respective UICC stages: **A** UICC I, **B** UICC II, **C** UICC III, **D** UICC IV. OS was defined as the time in months from the date of diagnosis to death from any cause. Cancer-specific mortality was not used; therefore, all-cause mortality served as the endpoint. Patients who were lost to follow-up were right-censored at the date of their last documented contact

**Fig. 2:**
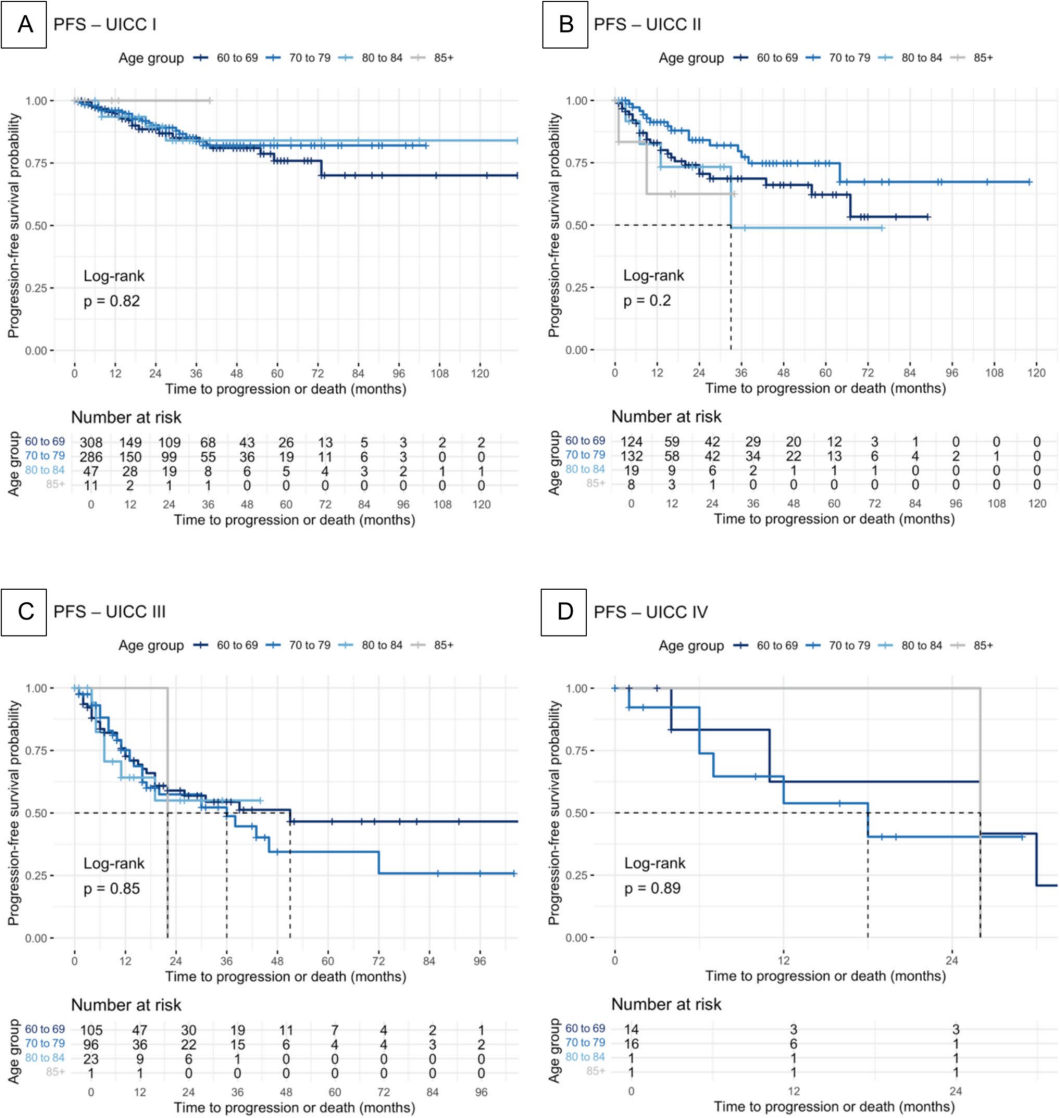
Kaplan–Meier progression-free survival analysis Kaplan–Meier survival curves were constructed to estimate univariate progression-free survival (PFS). Differences between groups were assessed using the log-rank test. PFS was analyzed for the different age groups in the respective UICC stages: **A** UICC I, **B** UICC II, **C** UICC III, **D** UICC IV. PFS was defined as the time in months from diagnosis to the first occurrence of disease progression. Progression was defined as any of the following: local recurrence, nodal recurrence, or systemic progression. Patients without a documented progression event were right-censored at their last follow-up

Kaplan–Meier survival analysis demonstrated a statistically significant difference in OS between elderly and younger patients with UICC I (*p* = 0.0032) and UICC II (*p* = 0.0079). No significant differences in OS were observed between UICC III and UICC IV. The Kaplan–Meier survival analysis in relation to PFS revealed no statistically significant disparities across all UICC tumor stages. Kaplan–Meier-Curves are represented in Fig. 1 and Fig. 2.

### Multivariate regression analysis

Multivariate Cox regression analysis showed significantly poorer overall survival for patients aged 80 or older compared to patients aged between 60 and 69 (HR = 2.05, *p* = 0.01; HR = 3.20 *p* = 0.02). There were no significant differences in overall survival regarding the first and second group. Female patients showed a significantly better OS than males (HR = 0.63, *p* = 0.01). Patients with NEC showed better OS than those with ACC (HR = 0.45, *p* = 0.04). Increasing tumor size in cm was significantly associated with poorer OS (HR = 1.08, *p* = 0.005). Additionally, patients with positive lymph node status had significantly poorer OS than N0 (HR = 1.8, *p* = 0.001), and patients who received adjuvant chemotherapy had a significantly better OS (HR = 0.45, *p* = 0.005). Higher CRP at staging was associated with poorer OS (HR = 1.04, *p* = 0.01).

PFS was not significantly associated with age. We found significant differences regarding NEC vs. ACC (HR = 0.39, *p* = 0.02), increasing tumor size in cm (HR = 1.16, *p* < 0.0001) and lymph node status (HR = 2.17, *p* < 0.0001). Metastases prior to the surgery (HR = 1.33, *p* < 0.0001) were significantly associated with poorer PFS. All results are shown in Table 4A and B.

**Table 4:** Results from Cox regression analysis of overall survival and overall progression-free survival

A	Cox Regression OS						
		coef	HR	se	*z*-value	*p*-value	
	70 to 79 vs. 60 to 69	0,23	1,26	0,18	1,29	0,20	
	80 to 84 vs. 60 to 69	0,72	2,05	0,27	2,65	0,01	**
	≥ 85 vs. 60 to 69	1,16	3,20	0,49	2,39	0,02	*
	female vs. male	−0,46	0,63	0,18	−2,50	0,01	*
	Charlson score	0,00	1,00	0,05	−0,06	0,95	
	NEC vs. ACC	−0,79	0,45	0,38	−2,09	0,04	*
	other vs. ACC	0,09	1,09	0,54	0,17	0,87	
	SCC vs. ACC	−0,24	0,78	0,18	−1,35	0,18	
	tumor size in cm	0,08	1,08	0,03	2,83	0,005	**
	N1N2N3 vs. N0	0,59	1,80	0,18	3,24	0,001	**
	M1 vs. M0	0,02	1,02	0,40	0,04	0,96	
	adjuvant CTx	−0,80	0,45	0,29	−2,81	0,005	**
	adjuvant RTx	0,42	1,53	0,32	1,31	0,19	
	neoadjuvant CTx	0,09	1,09	0,37	0,24	0,81	
	neoadjuvant RTx	0,02	1,02	0,67	0,03	0,98	
	FEV1%	0,00	1,00	0,00	−1,08	0,28	
	Hb at staging	−0,08	0,93	0,04	−1,71	0,09	
	creatinine at staging	0,10	1,10	0,14	0,73	0,47	
	CRP at staging	0,03	1,04	0,01	2,48	0,01	*
	leukocytes at staging	0,02	1,02	0,02	0,92	0,36	
B	Cox Regression PFS						
		coef	HR	se	*z*-value	*p*-value	
	70 to 79 vs. 60 to 69	−0,04	0,96	0,19	−0,22	0,82	
	80 to 84 vs. 60 to 69	0,11	1,12	0,30	0,37	0,71	
	≥ 85 vs. 60 to 69	0,78	2,18	0,55	1,43	0,15	
	female vs. male	−0,15	0,86	0,18	−0,85	0,40	
	Charlson score	−0,04	0,96	0,05	−0,85	0,40	
	NEC vs. ACC	−0,95	0,39	0,40	−2,37	0,02	*
	other vs. ACC	0,45	1,57	0,47	0,97	0,33	
	SCC vs. ACC	−0,28	0,76	0,20	−1,42	0,15	
	tumor size in cm	0,15	1,16	0,03	5,84	< 0.0001	***
	N1N2N3 vs. N0	0,77	2,17	0,19	4,01	< 0.0001	***
	M1 vs. M0	1,33	3,80	0,31	4,26	< 0.0001	***
	adjuvant CTx	−0,28	0,76	0,24	−1,15	0,25	
	adjuvant RTx	0,31	1,36	0,28	1,11	0,27	
	neoadjuvant CTx	0,44	1,55	0,34	1,30	0,19	
	neoadjuvant RTx	−0,37	0,69	0,67	−0,55	0,58	
	FEV1%	0,01	1,01	0,00	1,28	0,20	
	Hb at staging	−0,03	0,97	0,05	−0,58	0,56	
	creatinine at staging	−0,01	0,99	0,20	−0,07	0,95	
	CRP at staging	0,03	1,03	0,02	1,56	0,12	
	leukocytes at staging	0,01	1,01	0,03	0,40	0,69	

Results from Cox regression analysis of overall survival (A), progression-free survival (B) and age adjusted by sex, American Society of Anesthesiologist risk classification (ASA), Charlson comorbidity index, histological type, tumor size, lymph node involvement (N0N1N2N3), distant metastasis (M0M1), adjuvant chemotherapy (CTx), adjuvant radiotherapy (RTx), neoadjuvant chemotherapy, neoadjuvant radiotherapy, Forced Expiratory Volume in 1 s (FEV1), hemoglobin at staging, creatinine at staging, c-reactive protein (CRP) at staging and leukocytes at staging

*OS* overall survival, *PFS* progression-free survival, *coef* coefficient of variation, *HR* hazard ratio, *se* standard error, *z-value* standard value, *p-value* probability value, *NEC* neuroendocrine carcinoma, *ACC* adenocarcinoma, *SCC* squamous-cell carcinoma

## Discussion

Lung cancer is particularly common in older people, meaning that thoracic surgeons are increasingly faced with treating patients over the age of 80 with resectable NSCLC. In industrial states the median age of lung cancer is 71. It accounts for 12,2% of all new cancer cases and accounts for 20,8% of all cancer deaths [16].

Its frequent occurrence and increasing number in elderly patients demand the best possible therapy to extend patients' lives while also considering their quality of life. Surgery is the best option and should be the first line of therapy, if possible. However, the complication rate increases proportionally with age [8, 17, 18].

Unlike other previously published studies, this study was able to include a large number of patients, especially in the groups of patients being 70 or older [6, 8, 19–21]. In addition, many of the studies published to date are based on a single comparator group of older patients (mostly patients over 75 or 80 years of age) lacking a detailed age breakdown, especially in the very old age groups [6, 8, 11, 19–23]. We are convinced that it is essential to take a closer look at the very elderly, as their surgical treatment becomes increasingly important. Moreover, in contrast to previous studies, this research incorporates all operable tumor stages, a crucial element in the comprehensive assessment of treatment outcomes [6, 9, 11, 12, 24].

The combination of a large cohort, detailed division into age groups, and inclusion of all operable tumor stages provides us an excellent insight into the outcomes of surgical treatment in older patients.

With regard to tumor characteristics, we found virtually no differences between the groups. In particular, we found no significant differences in terms of UICC tumor stage or histological types. Therefore, their influence on the results is considered negligible, and all groups began the surgical treatment under similar tumor conditions. (Table 2 and S2). We were able to show that the older patients (≥ 80) had more comorbidities as evidenced by higher CCI, kidney insufficiency, cardiac impairment. However, there were no major differences in postoperative complications compared to the younger groups. The oldest age group (≥ 85) was only more likely to have cardiac arrythmias postoperatively and a longer hospital stay after surgery. Pie et al. reported three independent risk factors for postoperative complications – (a) pneumonectomy, which was not included in our study; (b) a prolonged surgical time, for which we could not find any evidence across the age groups in our cohort; and (c) a CCI of 3 or more was found to be an independent risk factor [20]. We found that the CCI was significantly increasing with age. However, this was not associated with higher postoperative complications, reduced OS or PFS.

The physical conditions of our oldest patients were superior to what could have been anticipated, considering their age. FEV1 was significantly lower in younger patients and increased with advancing age. Furthermore, the patients in group four (≥ 85) had a lower body mass index (BMI) and lower CCI compared to those in group three (80 to 84). Moreover, group four outperformed group three in terms of cardiac function, which was defined by coronary heart disease, atrial fibrillation and atrial hypertension. Previous findings from medical studies have indicated a positive correlation between age and the prevalence of comorbidities, as well as the risk of complications [6–8]. The results of our study therefore suggest that the older patients were carefully selected, which may have influenced the results and could have led to a possible limitation of the study. (Table 1 and S1, Table 3 and S3).

We found that biological age is more important than numerical age and that surgical treatment in elderly patients is not associated with more postoperative complications, higher operative risk or poorer outcome. If lung and heart function is not impaired and performance status is not severely compromised, there are no absolute contraindications for surgical treatment, even if patients ≥ 85 years. However, it is obvious that not every patient at an advanced age should be exposed to the risk of surgery.

The Kaplan–Meier survival analysis and multivariable cox regression further illustrate this point (Fig. 2 and Table 4). No statistically significant disparities in PFS were observed when the cohort was stratified according to the respective tumor stages. Unfortunately, we could not find many comparative studies on the PFS in elderly patients. Qiang et al. had similar results, although their inclusion criteria were slightly different [25].

OS was significantly poorer only for elderly patients in UICC I and UICC II compared to younger patients (Fig. 1A, B). There were no significant differences in OS regarding the other age groups and UICC stages (Fig. 1C, D). Multivariate Cox regression analysis showed significantly poorer overall survival for patients aged 80 or older compared to patients aged between 60 and 69. These findings in Kaplan–Meier survival analysis and multivariate Cox regression are understandable when one considers that most patients with small tumor stages have a similar life expectancy after surgery as the general population, and that young patients live longer than older patients. In advanced tumor stages, life expectancy is reduced due to the tumor disease, and age no longer plays a decisive role. In advanced tumors, life expectancy is similar across age groups, regardless of whether the patients are young or older.

A significantly higher proportion of patients in the younger age groups received neoadjuvant and adjuvant therapy. This was mainly due to a higher proportion of neoadjuvant and adjuvant chemotherapy.

One potential explanation for this phenomenon is the nephrotoxicity associated with chemotherapy [26]. In cases where renal insufficiency is documented, interdisciplinary tumor boards often exercise caution with regard to systemic therapy, opting for lower doses or refraining from its administration entirely. This observation potentially provides a rationale for the considerably lower incidence of chemotherapy observed in older age groups within the present study. Our data proved that kidney function worsens with age. Pre- and postoperative creatinine levels were significantly higher with increasing age. In addition, patients aged 80 to 84 showed significant moderate to severe kidney insufficiencies preoperatively compared to the other groups. Surgical intervention is a viable treatment option for patients with comorbidities, such as renal dysfunction, and may be the optimal treatment modality for these patients. Based on our findings, we recommend that interdisciplinary tumor boards consider surgical treatment, even for elderly patients.

The following limitations of this study must be taken into account. First, our study was retrospective and a single center study, so the results may have been affected by selection bias. The good physical performance of the very old patients in particular indicates that they were well selected preoperatively. Our data provided little information on very old patients in poor general health. To minimize bias, only lobectomies were considered. Patients who underwent wedge resections or segmental resections were predominantly those with severe comorbidities or significantly impaired lung function. Segment resection was not the standard procedure for operable patients with NSCLC, particularly not in the early years of the observation period when there was still insufficient knowledge about the prognosis after segment resection. Pneumonectomies in elderly and very elderly patients remain a rare procedure. The exclusion of these resection procedures has substantially enhanced the homogeneity of the cohort. Second, we did not analyze data of NSCLC patients who did not undergo surgical treatment. This means we had no comparable data on OS and PFS without surgical treatment. In further studies, a comparison between surgically treated and non-surgically treated patients should be carried out, if possible multicentrically.

## Conclusion

Surgical therapies can be applied to old and very old patients at any operable tumor stage and are often the best treatment option. A careful selection, especially in the very old patients, is crucial for the outcome. Lung and heart function as well as performance status should not be severely impaired in order to minimize the perioperative risk. The numerical age is just a reference point, while the patient’s biological age is more important.

## Supplementary Information

Below is the link to the electronic supplementary material.

Supplementary file1 (DOCX 19 KB)

Supplementary file2 (DOCX 18 KB)

Supplementary file3 (DOCX 17 KB)

## Data Availability

Data from 1680 patients were recorded in the database. This data is still being analyzed and further studies are in progress. In order not to jeopardize the data for the new studies, they cannot be disclosed at this time. We ask for your understanding. The data sets and materials of the current study are available on reasonable request from the corresponding author.
