# Supplementary material for: Is surgery with curative intent feasible in old and very old patients with non-small cell lung cancer? – Experience of a certified lung cancer center over one decade
Source: Langenbecks Arch Surg. 2026 Feb 26;411(1):92. doi: 10.1007/s00423-026-03995-7 (PMC12975854; doi:10.1007/s00423-026-03995-7)
Supplement: Supplementary file 1 — Supplementary file1 (DOCX 19 KB) [file 423_2026_3995_MOESM1_ESM.docx]

**Supplementary Table S1: Patient characteristics of study population**

|  |  |  |  |  |  |  |  |  |  |  |
| --- | --- | --- | --- | --- | --- | --- | --- | --- | --- | --- |
|  | 60 to 69 (n = 565) | | 70 to 79 (n = 545) | | 80 to 84 (n = 91) | | ≥85 (n = 21) | |  |  |
|  | n | % | n | % | n | % | n | % | p-value |  |
| sex |  |  |  |  |  |  |  |  |  |  |
| male | 318 | 56,3% | 307 | 56,3% | 56 | 61,5% | 14 | 66,7% |  |  |
| female | 247 | 43,7% | 238 | 43,7% | 35 | 38,5% | 7 | 33,3% | 0,62 |  |
| ASA |  |  |  |  |  |  |  |  |  |  |
| 1 | 10 | 1,8% | 3 | 0,6% | 0 | 0,0% | 0 | 0,0% |  |  |
| 2 | 144 | 25,5% | 116 | 21,3% | 18 | 19,8% | 4 | 19,0% |  |  |
| 3 | 336 | 59,5% | 369 | 67,7% | 65 | 71,4% | 17 | 81,0% |  |  |
| unknown | 75 | 13,3% | 57 | 10,5% | 8 | 8,8% | 0 | 0,0% | 0,06 | . |
| current smoker |  |  |  |  |  |  |  |  |  |  |
| yes | 178 | 31,5% | 88 | 16,1% | 10 | 11,0% | 1 | 4,8% |  |  |
| no | 374 | 66,2% | 440 | 80,7% | 80 | 87,9% | 20 | 95,2% | <0.0001 | *** |
| unknown | 13 | 2,3% | 17 | 3,1% | 1 | 1,1% | 0 | 0,0% |  |  |
| comorbidities |  |  |  |  |  |  |  |  |  |  |
| mild liver disease | 13 | 2,3% | 8 | 1,5% | 0 | 0,0% | 0 | 0,0% | 0,48 |  |
| diabetes without end-organ damage | 70 | 12,4% | 89 | 16,3% | 16 | 17,6% | 2 | 9,5% | 0,20 |  |
| diabetes with end-organ damage | 9 | 1,6% | 14 | 2,6% | 2 | 2,2% | 0 | 0,0% | 0,69 |  |
| dementia | 0 | 0,0% | 1 | 0,2% | 0 | 0,0% | 1 | 4,8% | 1,00 |  |
| gastroduodenal ulcer | 18 | 3,2% | 12 | 2,2% | 2 | 2,2% | 1 | 4,8% | 0,51 |  |

Patient characteristics of lung cancer patients older than 60 years with lobectomy. Absolute and relative frequency of categorical variables, n= number, p-value = probability value, ASA= American Society of Anesthesiologist risk classification
