# Supplementary material for: Is surgery with curative intent feasible in old and very old patients with non-small cell lung cancer? – Experience of a certified lung cancer center over one decade
Source: Langenbecks Arch Surg. 2026 Feb 26;411(1):92. doi: 10.1007/s00423-026-03995-7 (PMC12975854; doi:10.1007/s00423-026-03995-7)
Supplement: Supplementary file 2 — Supplementary file2 (DOCX 18 KB) [file 423_2026_3995_MOESM2_ESM.docx]

**Supplementary Table S2: Tumor characteristics of study population**

|  |  |  |  |  |  |  |  |  |  |
| --- | --- | --- | --- | --- | --- | --- | --- | --- | --- |
|  | 60 to 69 (n = 565) | | 70 to 79 (n = 545) | | 80 to 84 (n = 91) | | ≥85 (n = 21) | |  |
|  | n | % | n | % | n | % | n | % | p-value |
| location |  |  |  |  |  |  |  |  |  |
| middle lobe | 34 | 6,0% | 35 | 6,4% | 5 | 5,5% | 2 | 9,5% | 0,84 |
| upper lobe | 341 | 60,4% | 316 | 58,0% | 47 | 51,6% | 10 | 47,6% | 0,30 |
| lower lobe | 190 | 33,6% | 194 | 35,6% | 39 | 42,9% | 9 | 42,9% | 0,32 |
| side |  |  |  |  |  |  |  |  |  |
| left | 228 | 40,4% | 235 | 43,1% | 39 | 42,9% | 9 | 42,9% |  |
| right | 337 | 59,6% | 310 | 56,9% | 52 | 57,1% | 12 | 57,1% | 0,82 |
| location and side |  |  |  |  |  |  |  |  |  |
| lower left | 84 | 14,9% | 93 | 17,1% | 18 | 19,8% | 4 | 19,0% | 0,96 |
| lower right | 106 | 18,8% | 101 | 18,5% | 21 | 23,1% | 5 | 23,8% | 0,52 |
| middle right | 34 | 6,0% | 35 | 6,4% | 5 | 5,5% | 2 | 9,5% | 0,45 |
| upper left | 144 | 25,5% | 142 | 26,1% | 21 | 23,1% | 5 | 23,8% | 0,65 |
| upper right | 197 | 34,9% | 174 | 31,9% | 26 | 28,6% | 5 | 23,8% | 0,84 |

Tumor characteristics of lung cancer patients older than 60 years with lobectomy. Absolute and relative frequency of categorical variables, n= number, p-value = probability value
