# Supplementary material for: Is surgery with curative intent feasible in old and very old patients with non-small cell lung cancer? – Experience of a certified lung cancer center over one decade
Source: Langenbecks Arch Surg. 2026 Feb 26;411(1):92. doi: 10.1007/s00423-026-03995-7 (PMC12975854; doi:10.1007/s00423-026-03995-7)
Supplement: Supplementary file 3 — Supplementary file3 (DOCX 17 KB) [file 423_2026_3995_MOESM3_ESM.docx]

**Supplementary Table S3: intraoperative data and complications**

|  |  |  |  |  |  |  |  |  |  |
| --- | --- | --- | --- | --- | --- | --- | --- | --- | --- |
|  | 60 to 69 (n = 565) | | 70 to 79 (n = 545) | | 80 to 84 (n = 91) | | ≥85 (n = 21) | |  |
|  | mean | sd | mean | sd | mean | sd | mean | sd | p-value |
| blood loss in ml during operation | 432,0 | 311,0 | 447,0 | 387,0 | 476,0 | 399,0 | 412,0 | 172,0 | 0,96 |
| total volume resected lung in ml | 1517,0 | 852,0 | 1509,0 | 1056,0 | 1403,0 | 876,0 | 1463,0 | 917,0 | 0,35 |
|  | n | % | n | % | n | % | n | % | p-value |
| complications |  |  |  |  |  |  |  |  |  |
| peridural catheter | 258 | 45,7% | 257 | 47,2% | 42 | 46,2% | 11 | 52,4% | 0,85 |
| pulmonary embolism | 1 | 0,2% | 5 | 0,9% | 1 | 1,1% | 0 | 0,0% | 0,27 |
| delirium | 2 | 0,4% | 4 | 0,7% | 1 | 1,1% | 1 | 4,8% | 0,09 |
| repeated thoracic puncture/chest tube placement | 40 | 7,1% | 40 | 7,3% | 5 | 5,5% | 2 | 9,5% | 0,85 |
| postoperative bronchoscopy | 65 | 11,5% | 56 | 10,3% | 9 | 9,9% | 2 | 9,5% | 0,93 |
| blood transfusion | 17 | 3,0% | 25 | 4,6% | 2 | 2,2% | 3 | 14,3% | 0,09 |

Perioperative outcomes of lung cancer patients older than 60. Means with standard deviation of numerical variables and absolute and relative frequency of categorical variables, sd = standard deviation, p-value = probability value, n= number
